# Nitrate Levels in Rural Drinking Water in Belize

**DOI:** 10.5696/2156-9614-10.27.200904

**Published:** 2020-08-19

**Authors:** Danladi Chiroma Husaini, Andrea Enriquez, Theslyn Arzu, Kelcia Miranda, Denise Mossiah, Crystal Cardinez

**Affiliations:** Pharmacy Program, Department of Allied Health, Faculty of Health Sciences, University of Belize, Belmopan, Belize

**Keywords:** drinking water quality, nitrate, contamination, pollution, Belize

## Abstract

**Background.:**

Health issues have been associated with the consumption of high levels of nitrates in drinking water. Rural agricultural communities in Belize play a large role in the economic growth of the country. These communities obtain drinking water directly from the ground and may be susceptible to nitrate consumption and at risk of developing diseases associated with nitrates.

**Objectives.:**

The present study examined nitrate levels in Belize's rural water supply with the aim of assessing its suitability for human and livestock consumption. The study also provides baseline data for monitoring the concentration of nitrates to prevent public health hazards in Belize.

**Methods.:**

Forty-three (43) water samples from reservoirs, wells, vats, and standpipes were collected from 40 villages in Belize and analyzed for nitrates using the cadmium reduction method. Nitrates were detected with an Orion® AquaMate® 8000 UV-Vis spectrophotometer at 520 nm. The Belize Coastal Zone Management Authority and Institute laboratory analyzed all water samples for nitrates.

**Results.:**

Except for four water samples from four different sites, all analyzed water samples were found to contain nitrate levels below 10 mg/L. Nitrate levels above 10 mg/L were seen in a few samples in the northern part of the country, probably due to agricultural activities in these areas.

**Conclusions.:**

Belize's rural drinking water contains low levels of nitrates, except for a few villages where the levels exceeded the acceptable limit of 10 mg/L. Higher levels of nitrates detected in a few villages need regular evaluation and monitoring to avoid public health issues as well as prevent harm to livestock.

**Competing Interests.:**

The authors declare no competing financial interests.

## Introduction

Excessive consumption of nitrates has been shown to affect the oxygen-carrying capacity of blood, leading to methemoglobinemia, also known as blue baby syndrome.^1^ Serious illness and even death resulting from methemoglobinemia symptoms have been reported, including, among other symptoms, hypotension, stomach cramps, increased heart rate, and vomiting.^1^–^3^

The standard acceptable concentration of nitrates in drinking water is 10 mg of nitrate (measured as nitrogen) per liter of drinking water (mg/L).^4^ Drinking water with levels of nitrate at or below 10 mg/L is generally considered acceptable *(Supplemental Material—Table 1).* Whereas low levels of nitrates in drinking water can occur as a natural process, usually less than 3 mg/L, nitrate levels exceeding 10 mg/L are of concern to human health. Higher levels of nitrates from wastewater, animal feedlots, urban drainages, landfills, septic systems runoff, or leakage from fertilized soil have been reported to increase health burdens and are a threat to public health in many communities.^2^,^3^,^5^,^6^

Naturally occurring nitrates in surface and groundwater usually occur at levels that do not generally cause health concerns. However, overuse of chemical fertilizers for agricultural purposes, improper disposal of animal and human waste, well location, or improper well construction can increase the levels of nitrates in well water, often leading to an increased concentration of nitrates in humans when such water is consumed.^7^

Furthermore, animal feedlots, fertilizers, septic systems, food processing waste, and industrial wastes are all sources of nitrates that can contaminate well water in both rural and urban areas. These can all result in increased human consumption of nitrates, thereby increasing the risks for nitrate-induced diseases.^3^, ^8^–^10^

**Figure 1:**
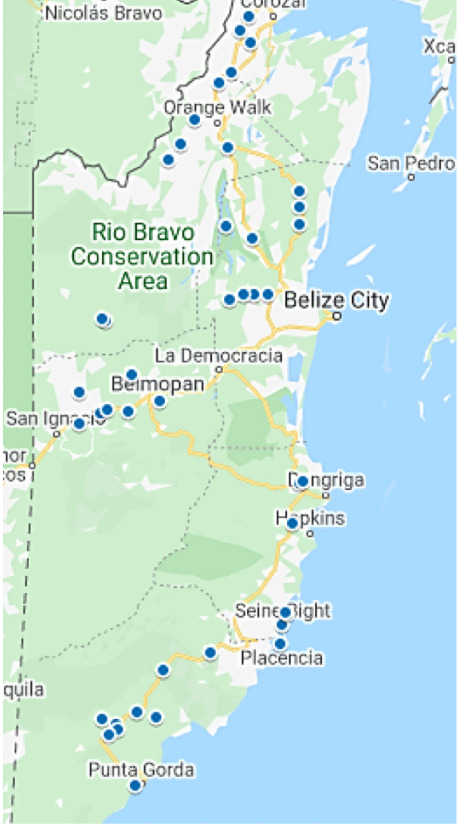
Map of Belize showing rural water supply system sample collection sites. Source: Google maps

**Figure 2:**
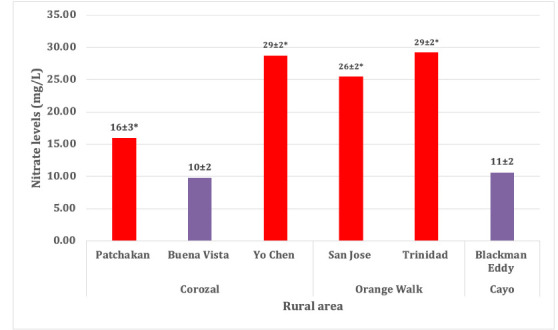
Nitrate levels in rural drinking water that require monitoring

Belize's Ministry of Health reported that about 97% of Belize's population has access to safe water.^11^ Presently, most cities, towns, and some villages are supplied potable water by Belize Water Services Limited, while several villages use a ‘rural water supply' system.^12^ Rural water supply systems usually have a village water board appointed every three years and are tasked with the responsibility of supplying water to villages not supplied by Belize Water Services Limited. Village water boards do this by directly pumping water to overhead concrete reservoirs *(Supplemental Material—Figure 1)* that supply water directly to homes. Rural water supply systems also provide water boreholes (water standpipes) *(Supplemental Material—Figure 2)* to some of the communities that lack a reservoir water supply.

**Figure 3:**
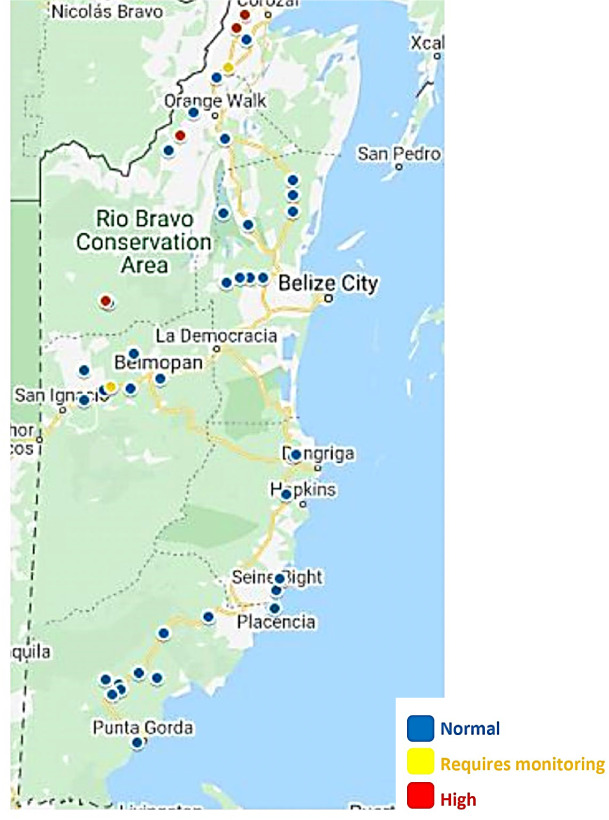
Map of water collection sites indicating nitrate levels. Source: Google maps

Residents using such water services are responsible for the treatment of their water on a monthly basis, with assistance from the government. Selection of the village water board members is highly politicized, and the board is almost always inadequately funded and there are many challenges involved in carrying out its functions. In many instances, water is not properly treated but pumped directly for consumption, ignoring international guidelines for drinking water with the possibility of risks to public health.^13^–^15^ Furthermore, village water board systems are not equipped to conduct tests for nitrates and other toxic metals found in water, making consumers more vulnerable to the risk of diseases associated with environmental contaminants. In some communities as well as many homes within these villages, many have improvised other means for water supply and storage, such as wells. Water obtained directly from wells is susceptible to nitrate contamination due to the overuse of chemical fertilizers for agricultural purposes, improper disposal of animal and human waste, and well location or improper well construction can increase the levels of nitrates in well water, often leading to increased concentrations of nitrates in humans when such water is consumed.^2^,^3^ It is not an uncommon practice, therefore, for some homes in Belize's rural communities to store water in cement *(Supplemental Material—Figure 3)* or plastic *(Supplemental Material— Figure 4)* vats for cooking, laundry, bathing, feeding livestock, and drinking. At other times, rainwater is directly collected into vats for these purposes during rainy seasons. In order to purify water for drinking, some villagers use mechanical filters, chemical disinfectants (e.g., chlorine), or may boil water. Although these methods can kill bacteria and address other impurities, these processes may not necessarily remove nitrates from water, and in fact, the boiling process can actually increase nitrate levels.^16^

Despite the government's efforts at providing potable water supply to the entire country, many villages still use rural water supply systems as a source of drinking water. Many villages in Belize are also involved in small or large-scale farming activities. Nitrate contamination of drinking water in rural areas could arise from a lack of proper water treatment processes, the use of fertilizers on farms, proximity of farms to wells, proximity of septic tanks to residential areas, and rainforest terrain. Through these sources, nitrates have been reported to leach into open or underground water supplies and can be a source of contamination through water consumption.^16^ To the best of our knowledge, there is little data and information on nitrate levels in rural drinking water in Belize. Moreover, while the government of Belize is doing its best to provide safe potable water to the public, there is no national water board or commission to effectively monitor, regulate, and standardize water management in the country.

Although presently no studies in Belize have shown a direct link between nitrates and associated diseases, these diseases are prevalent in some communities. The increased nitrate burden in underground drinking water needs to be assessed in order to protect humans and livestock from pollution. The direct consumption of unprocessed water by villagers, agricultural activities in villages that may employ the use of fertilizers, as well as the lack of a national water regulating board to ensure standardization in potable water supply necessitates the need for the current study. The present study aims to assess the level of nitrates in rural potable water as well as evaluate its suitability for human and livestock consumption. The study also provides baseline data for a more extensive study, and for monitoring nitrates in rural communities without a purified water supply in Belize.

## Methods

Belize has subtropical weather with clearly defined seasons. The rainy season is between the months of May and November, and the dry season between the months of December and April. Intermittent rains are usually experienced during dry seasons, which create some variation in the wet and dry seasons. Although Belize is geographically located in Central America, it also a member of the Caribbean trade union. Agriculture and human settlements make about 20% of Belize's land usage, while more than 60% of the land is still made up of forest.^17^ Agriculture—and agro-based industries—form part of Belize's economy, with sugar and banana being the main sources of agricultural exports and foreign income.^18^ Belize is divided into six districts made up of Belize (east), Cayo (west), Corozal and Orange Walk (north), and Stann Creek and Toledo in the south. Although the populations of these districts differ, each has a sizable number of communities using rudimentary water supplies as a source of potable water.

### Study area

The study began by identifying villages that are presently using rudimentary water supplies or other sources of potable water apart from Belize Water Services Limited. A total of 109 communities using rural water supply systems were identified.^11^ Out of the 109 sites, 40 representative villages were selected and visited for sample collection *(Figure 1).* Samples from these sites were collected over a period of eight months (between April to November 2019). The first batch of samples were collected at the beginning of the rainy season from April to early June, while the second batch of the samples were collected during the peak of the rainy season between July to early November. Water samples from the Belize Water Services Limited water supply system were not collected for analysis in the present study. Only water from the rural water supply system was collected from villages in each district with a rudimentary water supply system. The villages were randomly selected from the list of all villages in the district with a rudimentary water supply.

### Water sample collection and analysis

A total of two water samples were collected from reservoirs, wells, vats, and standpipes from 40 villages using plastic bottles as shown in *Supplemental Material—Figure 5*. Plastic bottles were used to collect 43 water samples from 40 villages across all of the districts of Belize as shown in Figure 1. New 1000 mL bottles were purchased from Bowen & Bowen Limited^®^, thoroughly rinsed with purified water, dried, and used for water sample collection. Before sample collection, the bottles were also thoroughly rinsed with the respective water samples at the site of collection. Fifteen (15) water samples were collected from reservoirs, ten from wells, three from standpipes (boreholes), and 15 from vats *(Supplemental Material—Figure 5).* Water samples were collected from each district. Nine water samples were collected each from Belize and Toledo districts, seven each from Cayo, Stann Creek, and Orange Walk districts, and four from Cayo district. Two water samples were collected from either a reservoir, a vat, a standpipe, a well, or both, depending on what was available in the village. Collected water samples were immediately transported to the laboratory of the Belize Coastal Zone Management Authority and Institute and analyzed for nitrates. Water samples that were not immediately analyzed were filtered and refrigerated below 4°C until analysis. Testing and analysis were performed according to the United States Environmental Protection Agency (USEPA) for nitrate-nitrite analysis procedures.^14^ For quality assurance, each sample was analyzed twice, and the results were statistically computed and presented.

### Ethical approval

This study did not pose any risk to humans and ethical approval for the research was not required. Although not required, permission to conduct the research was obtained from the Faculty of Health Sciences, University of Belize. Strict ethical principles were observed during the entire research process.

### Cadmium reduction method

The cadmium (Cd) reduction method was used to test nitrates in rural water samples. The mechanism through which Cd reduces nitrates to nitrites is represented by the following Equation 1:

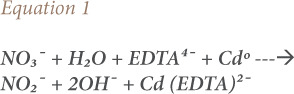
Reduced nitrite is formed from nitrate when Cd metal is added. A reaction of nitrite ions to form an immediate diazonium salt is seen in an acidic medium with sulfanilic acid.^19^ Gentisic acid then couples with the salt to form an amber-colored solution that can be measured using a spectrophotometer at a wavelength of 500 nm or 520 colorimeter.^20^ To reduce nitrate to nitrite, a filtered sample was passed through a column containing granulated copper Cd. Both the original nitrate in the sample and the reduced nitrite were then determined by diazotizing with sulfanilamide to form a highly colored azo dye in combination with N-(1-naphthyl)-ethylenediamine dihydrochloride. The intensity of the highly colored azo dye was then measured using an Orion^®^ AquaMate® 8000 UV-Vis spectrophotometer at 520 nm.^14^,^20^

### Statistical analysis

One-way analysis of variance testing was performed to compare the sets of data obtained from the different sites. The difference among the means analyzed by one-way analysis of variance was further analyzed using Student's t-test. A value of p ≤0.05 was considered statistically significant. Data are presented as the arithmetic mean ± SD. Estimated nitrates levels in water samples were compared with the USEPA acceptable safe drinking water levels.^8^,^9^,^11^

## Results

Detailed results of descriptive statistical analysis conducted on water samples from different rural settings in Belize are presented in the Supplemental Material. Nitrate concentration distributions from 40 sites were: standpipes (3) 0.5 – 4.3 mg/L, well water (10) 0 – 8 mg/L, vats (15) 0 – 11 mg/L and reservoirs (15) 1.2 – 29 mg/L, as shown in supplemental material *(Supplemental Material—Tables 2–7).* Low nitrate concentrations were observed in most of the tested water samples from rural communities in Belize.

In Belize district for instance, nitrate was not detected in water samples collected in the vat at Burrell Boom and Bermudian Landing well samples. The levels of nitrate seen in all the samples from the Belize districts were low and within acceptable safe levels. No significant differences were observed in all of the samples tested *(Supplemental Material—Table 2).* In the Cayo district, nitrate was detected 11±2 mg/L in vat water samples from Blackman Eddy village. Although the nitrate level at Blackman Eddy village was found to be significantly (p <0.05) higher when compared with other water samples in the district, the level detected was only slightly above the EPA acceptable level *(Supplemental Material—Table 3).* In the Corozal district however, all water samples collected were from reservoirs. Nitrate detected at Patchakan and Yo Chen reservoirs was significantly (p <0.05) higher and exceeded the acceptable limits for drinking water (*Figure 2 and Supplemental Material—Table 4*).

The detected levels of nitrates seen in water samples in the Orange Walk district are presented in Supplemental Material Table 5. Except for reservoir samples from the San Jose (26 mg/l) and Trinidad (29 mg/l) communities, where nitrate levels were significantly (p <0.05) higher and above safe levels, all other samples were within acceptable safe limits *(Figure 2 and Supplemental—Figure 6).* Nitrate levels from wells, vats, and reservoirs in Stann Creek *(Supplemental Material— Table 6)* and Toledo *(Supplemental Material—Table 7)* districts were all low and within safe limits. All of the significantly elevated nitrate levels were collected from reservoirs *(Supplemental Material—Figure 6).* All the water samples from standpipes *(Supplemental Material—Figure 7)* and wells *(Supplemental Material—Figure 8)* showed acceptable levels of nitrates below 10 mg/L in all the communities studied. Only one vat water sample (Blackman Eddy) showed levels of nitrates slightly above 10 mg/L *(Supplemental Material—Figure 9).* The nitrate level seen at Blackman Eddy was significantly higher when compared with samples from the same district, but was within the EPA acceptable limit. However, monitoring of nitrates at the site (Blackman Eddy) is recommended *(Figure 2).*

## Discussion

Except for a few rural communities, low nitrate levels were observed from drinking water samples in most rural communities in the present study *(Figure 3).* Nitrates at this level are generally considered safe for all usages.^4^ Nitrate levels from sites in the Cayo district were low and present no urgent need for monitoring (other than Blackman Eddy), except if agricultural activities or other sources of nitrates are increased in these areas *(Figure 2).*

Although nitrate levels between 11–40 mg/L are considered safe for short-term use by healthy adults, long-term use presents a risk for all populations.^4^,^11^,^21^ The levels of nitrates detected at some sites in Corozal were not safe for babies or for women who are pregnant or planning to become pregnant. Furthermore, the detected levels of nitrates at these sites are not safe for long-term consumption by livestock. Nitrate poisoning has been reported to be more pronounced in cattle and sheep because of their ability to convert nitrate to nitrite in their digestive tract.^7^,^22^ Since these communities are actively involved in small-scale livestock farming, the levels of nitrate detected may pose a risk to livestock if they regularly consume affected water. The water supply in these communities *(Figure 2),* therefore, needs to be treated and monitored for nitrates to avoid livestock contamination, and by extension poses a public health hazard to humans.^2^,^3^ Nitrate levels detected at Concepcion in the Corozal district was low and considered safe for human consumption.^4^

The discrepancies observed in water samples from the reservoir and vat samples at San Jose in the Orange Walk district could possibly be due to collected rainwater sometimes stored in vats by the residents, and not necessarily water directly from the reservoir or the wells. Naturally occurring nitrates in surface and groundwater are usually at a level that does not generally cause health issues. However, overuse of chemical fertilizers for agricultural purposes and improper disposal of animal and human waste, animal feeds, well location, or improper well construction can increase the levels of nitrates in groundwater, often leading to increased concentrations of nitrates in humans when such water is consumed.^3^ The high levels of nitrates seen in these communities could be attributed to similar activities possibly leading to an increased concentration of nitrates in humans when such water is consumed.^3^,^7^,^9^

All the water samples that showed significantly higher nitrate levels were from reservoirs *(Figure 2 and Supplemental—Figure 6).* Higher nitrate levels detected at Patchakan (16 mg/L), San Jose (26 mg/L), Yo Chen (29 mg/L), and Trinidad (29 mg/L) came from reservoirs *(Figure 2).* Since underground water is pumped directly into the reservoir in these communities, agricultural or other activities might contribute to the nitrate burden and the possibility of pollution when such water is used for drinking water or for livestock. In addition, these communities are heavily involved in sugar cane farming and other agricultural businesses, and application of nitrogen fertilizers might be considered to be a factor in the high level of nitrates seen at these sites. Most applied nitrogen fertilizers from agricultural activity eventually drain nitrogen and contaminate either surface or ground water, thereby increasing the concentrations of nitrites in water.^7^,^23^ The levels of nitrate detected at these sites call for further evaluation and monitoring to lower the risk to public health in these communities.

The dynamics of increased endogenous nitrosation and a combination of high nitrate intake have been reported to have a strong relationship with the development of diseases in humans and other animals.^2^ Several studies have shown relationships between disease development and nitrate consumption.^2^ Diseases such as colorectal cancers, thyroid disorders, colon and rectum cancers, and birth defects have all been associated with the consumption of high nitrate levels (beyond 10 mg/L), especially from drinking water.^2^,^24^–^27^ The risk of developing thyroid cancers from nitrates is a result of the competitive uptake of iodine. Iodine is required for normal thyroid function. An increase in the release of thyroid stimulating hormone resulting from a reduction in triiodothyronine and thyroxin due to nitrates make individuals susceptible to thyroid cancer development.^2^,^26^,^28^

The analysis of nitrates is a regular part of governmental policies in many countries where nitrates have been well researched and their adverse health effects have been documented. In the United States, for instance, comprehensive monitoring and analysis of nitrates in potable water are a part of every state's policy to ensure the provision of a safe public water supply.^2^,^29^ In Europe, the European Union Nitrates Directive called for the implementation and reporting of nitrates in drinking water.^30^,^31^ These directives and policies provided a framework for monitoring water and ensuring safe drinking water in member countries.^2^ Unfortunately, in Belize, policies on comprehensive monitoring and analysis of nitrates in rural drinking water is nonexistent, and consumption of nitrate-contaminated water could expose rural dwellers to nitrates and associated diseases.

Since people who consume contaminated water are more susceptible to developing various diseases and a strong relationship exists between disease, poverty, health, and access to clean potable water, regular water safety checks are needed to reduce morbidity and mortality and improve public health.^2^,^3^ The results of the current study point to the need for strategic promulgation and implementation of policies to monitor nitrate levels in public drinking water and the development of educational campaigns to create public awareness of the dangers of nitrates consumption above acceptable levels. Provision of water uncontaminated by nitrates and other metals as well as village water treatment facilities will help to curb the likelihood of harm due to consumption of water with high nitrate concentrations by rural dwellers in Belize.

### Limitations

A few limitations should be considered in the interpretation of the results of the present study. A lack of clearly defined seasonal variation during sample collection, and the limited number of samples collected for the study present some limitations. However, these limitations did not overshadow the strengths of the current study. To the best of our knowledge, this is the first published countrywide research on nitrates in the country of Belize. Additionally, the study made every effort to obtain representative water samples from all districts across Belize. In addition, the objective of the present study was to provide national baseline data for the development of future large-scale research in the field.

## Conclusions

The results of the present study provide baseline data for monitoring changes in nitrate levels in rural water supply systems in the country of Belize. Only four samples showed significantly high nitrate levels from across the 40 study sites. All other rural water supply system samples showed low levels of nitrate concentrations below the USEPA maximum acceptable concentration. The low concentrations of nitrates in the present study do not constitute immediate risks to public health. The results suggest a lack of surface and underground water contamination in the majority of studied rural communities. The four communities that showed higher levels of nitrates need regular evaluation and monitoring to avoid public health risks as well as prevent harm to livestock. The formulation of an independent water commission to monitor and regulate potable rural water supply systems is needed to ensure the provision of a safe water supply to these communities.

## References

[1] (2015). ToxFAQsTM for nitrate and nitrite [Internet].

[2] Ward MH, Jones RR, Brender JD, de Kok TM, Weyer PJ, Nolan BT, Villanueva CM, van Breda SG (2018). Drinking water nitrate and human health: an updated review. Int J Environ Res Public Health [Internet].

[3] Mehri F, Heshmati A, Moradi M, Khaneghah AM (2019). The concentration and health risk assessment of nitrate in vegetables and fruits samples of Iran. Toxin Rev [Internet].

[4] Ground water and drinking water: national primary drinking water regulations: inorganic chemicals [Internet].

[5] (2013). ATSDR case studies in environmental medicine nitrate/nitrite toxicity [Internet].

[6] van Duijvenbooden W, Matthijsen AJ (1989). Integrated criteria document nitrate [Internet].

[7] Chiroma HD, Ayinla GT, Orish OE, Emmanuel OO, Jigam AA, Makun HA, Sani GM, Johnson AO, Humphrey P (2007). Seasonal nitrate content of stream water, soil and some foodstuffs samples in Abuja municipal area of Federal Capital Territory, Nigeria. J Health Sci [Internet].

[8] Speijers GJ, van den Brandt PA (2003). Nitrite (and potential endogenous formation of N-nitroso compounds). In: Safety evaluation of certain food additives [Internet].

[9] Speijers GJ, van den Brandt PA (2003). Nitrate (and potential endogenous formation of N-nitroso compounds). In: Safety evaluation of certain food additives [Internet].

[10] Nitrate and drinking water from private wells [Internet].

[11] Grau J, Rihm A (2013). Water and sanitation in Belize [Internet].

[12] Belize Water Services: about us.

[13] (2011). Guidelines for drinking-water quality [Internet].

[14] Water: monitoring and assessment: 5.7 nitrates [Internet].

[15] (2010). Progress on sanitation and drinking-water: Joint Monitoring Programme 2010 update [Internet].

[16] Oram B (c2014). Nitrates and nitrites in drinking water groundwater and surface waters.

[17] Cherrington EA, Hernandez BE, Trejos NA, Smith OA, Anderson ER, Flores AI, Garcia BC (2010). Technical report: identification of threatened and resilient mangroves in the Belize barrier reef system.

[18] (2019). Annual report: 2018–19 [Internet].

[19] Dorich RA, Nelson DW (1984). Evaluation of manual cadmium reduction methods for determination of nitrate in potassium chloride extracts of soils. Soil Sci Soc Am J [Internet].

[20] Eaton AD, Clesceri LS, Rice EW, Arnold E, Greenberg AE, Franson MA (2005). Standard methods for the examination of water and wastewater.

[21] (2020). How can you remove nitrates in tap water [Internet].

[22] Block J (2020). Nitrate poisoning of livestock.

[23] Davidson EA, David MB, Galloway JN, Goodale CL, Haeuber R, Harrison JA, Howarth RW, Jaynes DB, Lowrance RR, Nolan BT, Peel JL, Pinder RW, Porter E, Snyder CS, Townsend AR, Ward MH (2012). Excess nitrogen in the U.S. environment: trends, risks, and solutions. Issues Ecol [Internet].

[24] Schullehner J, Hansen B, Thygesen M, Pedersen CB, Sigsgaard T (2018). Nitrate in drinking water and colorectal cancer risk: a nationwide population-based cohort study. Int J Cancer [Internet].

[25] Gatseva PD, Argirova MD (2008). High-nitrate levels in drinking water may be a risk factor for thyroid dysfunction in children and pregnant women living in rural Bulgarian areas. Int J Hyg Environ Health [Internet].

[26] Ward MH, Kilfoy BA, Weyer PJ, Anderson KE, Folsom AR, Cerhan JR (2010). Nitrate intake and the risk of thyroid cancer and thyroid disease. Epidemiology [Internet].

[27] De Roos AJ, Ward MH, Lynch CF, Cantor KP (2003). Nitrate in public water supplies and the risk of colon and rectum cancers. Epidemiology [Internet].

[28] Ward MH, deKok TM, Levallois P, Brender J, Gulis G, Nolan BT, VanDerslice J (2005). Workgroup report: drinking-water nitrate and health--recent findings and research needs. Environ Health Perspect [Internet].

[29] USGS water data for the nation [Internet].

[30] European Union (EU). Council Directive 91/676/EEC of 12 December 1991 Concerning the Protection of Waters against Pollution Caused by Nitrates from Agricultural Sources. https://eur-lex.europa.eu/legal-content/EN/ALL/?uri=CELEX%3A31991L0676.

[31] The nitrates directive [Internet].

