# Supplementary material for: Nitrate Levels in Rural Drinking Water in Belize
Source: J Health Pollut. 2020 Aug 19;10(27):200904. doi: 10.5696/2156-9614-10.27.200904 (PMC7453821; doi:10.5696/2156-9614-10.27.200904)
Supplement: Supplementary file 1 [file Husaini_Supplemental.docx]

**Supplemental Material**

**Table 1. Recommendations for nitrate consumption**


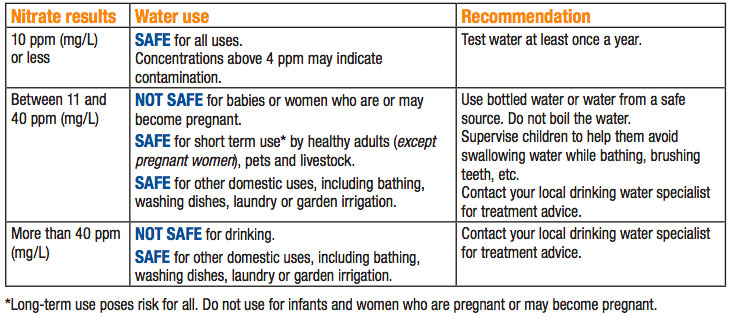


Source: Oregon Health Authority.**^21^**

| **Table 2. Water samples from the Belize district** | | |
| --- | --- | --- |
| **Site** | **Water source** | **Nitrates level (mg/l)** |
| Biscayne | Reservoir | 2.4±0.1 |
| Double Head Cabbage | Reservoir | 1.2±0 |
| Maskall | Reservoir | 4.4±1 |
| Santana | Reservoir | 4±0.4 |
| Burrell Boom | Vat | 0.00 (not detected) |
| Lucky Strike | Vat | 4±1 |
| Scotland Halfmoon | Vat | 4±1 |
| Bermudian Landing | Well | 0.00 (not detected) |
| Crooked Tree | Well | 3±0.01. |
| Values are expressed as mean ± SD for two samples. | | |

Four reservoir water samples, three vat and two well water samples were collected from Belize district. Nitrates were not detected in water samples from two sites. All water samples were below the acceptable limits.

| **Table 3. Water samples from Cayo district** | | |
| --- | --- | --- |
| **Site** | **Water source** | **Nitrates level (mg/l)** |
| Teakettle | Standpipe (borehole) | 2±0.1 |
| United Ville | Standpipe (borehole) | 1±0.2 |
| Blackman Eddy | Vat | 11±2* |
| Central Farm | Vat | 1±0.4 |
| Spanish Lookout | Vat | 2±0.3 |
| Valley of Peace | Vat | 8±1 |
| Salvapan | Well | 1±0.2 |
| Values are expressed as mean ± SD for two samples.  *Values significantly p <0.05 (student’s t-test) differ when compared to other values. | | |

For the Cayo district, one well water sample, two standpipe (borehole) water samples, and four vat water samples were collected. Vat water samples from Blackman Eddy require monitoring.

| **Table 4. Water samples from the Corozal district** | | |
| --- | --- | --- |
| **Site** | **Water source** | **Nitrates level (mg/l)** |
| Concepcion | Reservoir | 7±1.0 |
| Buena Vista | Reservoir | 10±2 |
| Patchakan | Reservoir | 16±3* |
| Yo Chen | Reservoir | 29±2* |
| Values are expressed as mean ± SD for two samples.  *Values significantly p <0.05 (student’s t-test) differ when compared to other values. | | |

In Corozal District, all the samples collected were from reservoirs. Reservoir water samples from Patchakan and Yo Chen were higher than 10 mg/L. Water samples from Buena Vista village require monitoring.

| **Table 5. Water samples from the Orange Walk district** | | |
| --- | --- | --- |
| **Site** | **Water source** | **Nitrates level (mg/l)** |
| August Pine Ridge | Reservoir | 7±0.2 |
| San Jose | Reservoir | 26±2* |
| San Jose | Vat | 3±1 |
| San Pablo | Reservoir | 7.1±1 |
| Carmelita | Vat | 5±0.1 |
| Trinidad | Reservoir | 29±2.0* |
| Yo Creek | Reservoir | 5±0.01 |
| Values are expressed as mean ± SD for two samples.  *Values significantly p <0.05 (student’s t-test) differ when compared to other values. | | |

Five reservoir water samples and two vat water samples were collected from the Orange Walk district. Except for San Jose and Trinidad communities, water samples from all other villages were low and within acceptable limits.

| **Table 6. Water samples from the Stann Creek district** | | |
| --- | --- | --- |
| **Site** | **Water source** | **Nitrates level (mg/l)** |
| Hope Creek | Reservoir | 2±0.02 |
| Hope Creek | Well | 2.3±1.1 |
| Placencia | Vat | 3±0.1 |
| Maya Beach | Vat | 4.4±1.1 |
| Seine Bight | Vat | 5±0.1 |
| Silk grass | Vat | 1±0.02 |
| Maya Beach | Standpipe (borehole) | 4.3±0.2 |
| Values are expressed as mean ± SD for two samples. | | |

Seven samples comprising of one reservoir, one well, (borehole), and four vats were collected from the Stann Creek district. Low nitrate levels were detected in all water samples from the district.

| **Table 7. Water samples from the Toledo district** | | |
| --- | --- | --- |
| **Site** | **Water source** | **Nitrates level**  **(mg/l)** |
| Humming Bird | Reservoir | 8±1 |
| Golden Stream | Vat | 4.4±0.3 |
| Silver Creek | Vat | 5.3±1 |
| Big Fall | Well | 4.4±1 |
| Dumb Well | Well | 3±0.3 |
| Hicatee | Well | 8±1 |
| Indian Creek | Well | 4±1 |
| Medina Bank | Well | 5.2±0.1 |
| San Miguel | Well | 3.1±0.4 |
| Values are expressed as mean ± SD for two samples. | | |

A total of nine samples were collected from the Toledo district: one sample from a reservoir, two from the vat, and six from wells.

**
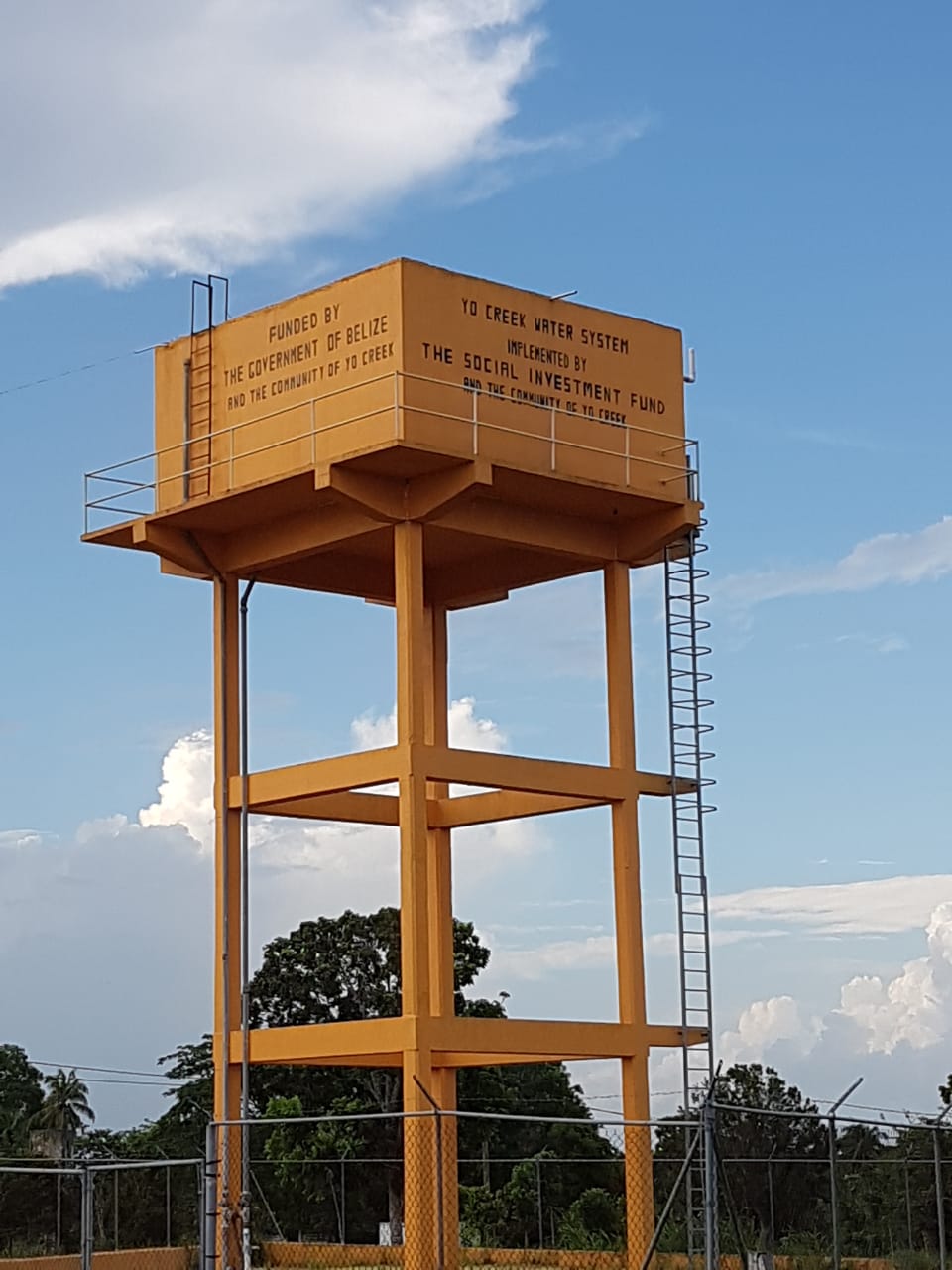
**

**Figure 1. Reservoir**

Photo by author

**
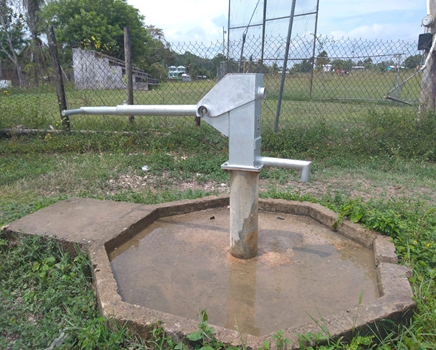
**

**Figure 2. Standpipe** (Borehole)

Photo by author

**
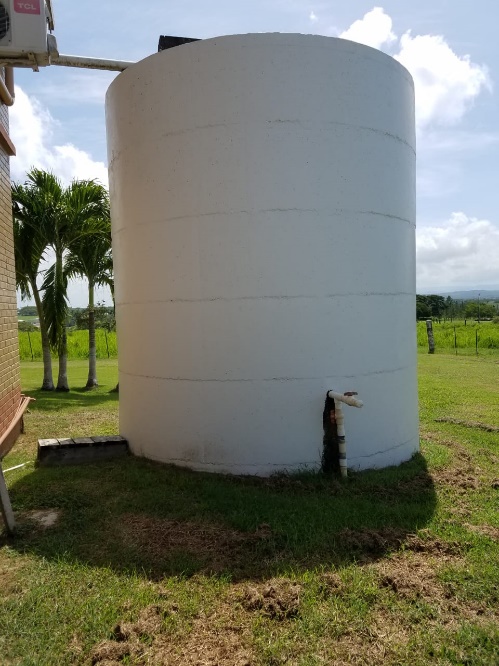
**

**Figure 3. Cement vat**

Photo by author

**
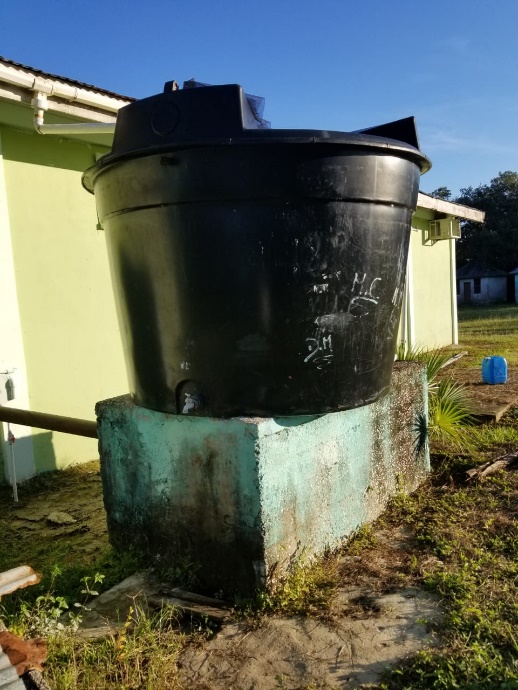
**

**Figure 4. Plastic vat**

Photo by author

**Figure 5. Sources of water samples**

Samples were analyzed twice, statistically computed and presented.

**Figure 6. Nitrate levels in reservoir water samples**

**Figure 7. Water samples from standpipes (boreholes)**

**Figure 8. Nitrate levels in well water samples**

**Figure 9. Nitrate levels in vat water samples**
